# Quality of Life after Transcatheter Arterial Chemoembolization Combined with Radiofrequency Ablation in Patients with Unresectable Hepatocellular Carcinoma Compared with Transcatheter Arterial Chemoembolization alone

**DOI:** 10.31557/APJCP.2021.22.4.1255

**Published:** 2021-04

**Authors:** Taha M Hassanin, Yasser Fouad, Alshymaa Hassnine, Mohamad Eisawy, Naglaa Farag, Wael Abdel Ghany

**Affiliations:** 1 *Department of Endemic Medicine and Gastroenterology, Faculty of Medicine, Minia University, Egypt. *; 2 *Department of Radiology Faculty of Medicine, Minia University, Egypt. *; 3 *Department of Clinical Pathology, Faculty of Medicine, Minia University, Egypt.*

**Keywords:** Quality of life, hepatocellular carcinoma, transarterial chemoembolization, radio frequency ablation

## Abstract

**Aim:**

The aim of this study was to assess quality of life (QoL) in patients with unresectable hepatocellular carcinoma (HCC) after transcatheter arterial chemoembolization (TACE) compared to TACE plus radiofrequency ablation (RFA) done at the same sitting, and to assess tumor therapy response after these 2 palliative interventions.

**Methods:**

73 patients with unresectable HCC (BCLC-B) were included. Patients with tumor ≤ 5 cm were subjected to TACE (N = 45) while patients with tumors > 5 cm were subjected to TACE followed immediately by RFA (N = 28). QoL was evaluated with two validated questionnaires (EORTC QLQ-30 and EORTC HCC18). These questionnaires were filled out before intervention, 2 weeks and 2 months after intervention. Pre/post interventional changes were analyzed. The modified response evaluation criteria in solid tumor (mRECIST) were employed for the evaluation of therapeutic efficacy.

**Results:**

Baseline global health status/QoL was significantly higher in TACE group (64.1%) compared to TACE-RFA group (51.2%). Two weeks after intervention: the absolute decrease in global health state was higher in TACE-RFA (- 12.1%) compared to TACE (- 6.3%, p = 0.411). Less impairment was found in TACE group compared to TACE-RFA group for physical/social functioning, fatigue and pain but it was statistically insignificant. Two months after intervention; TACE-RFA group showed significant improvement in global health score, social and physical functioning scores, as well as significant improvement in pain and fatigue compared to TACE group. The therapeutic efficacy of TACE-RFA was better than TACE alone: complete remission, partial remission, stable disease and progressive disease were 17.9%, 32.1%, 42.9% and 7.1% Vs11.1%, 22.2%, 48.9% and 17.8%, respectively).

**Conclusion:**

Neither TACE nor TACE-RFA showed a significant decrease in QoL in patients with unresectable HCC two weeks after intervention. However, two months after intervention; TACE-RFA showed significant improvement in global health score compared to TACE monotherapy. TACE-RFA appeared safe, effective and more favorable than TACE monotherapy.

## Introduction

Hepatocellular carcinoma (HCC) is the most common primary liver cancer, representing the sixth leading cause of cancer and the third leading cause of cancer-related mortality (Raoul et al., 2019). Curative therapies like surgical resection or liver transplant are not feasible for most HCC patients as they are often diagnosed when the disease is advanced. Radiofrequency ablation (RFA) and transarterial chemoembolization (TACE) are used in these patients to prolong life and/or alleviate the cancer symptoms but the overall survival remains poor (Shipra et al., 2014). RFA has been widely approved as a therapeutic modality for small HCC (Sangiovanni et al., 2004; Torre et al., 2015). The complete response induced by RFA in such cases can provide a survival rate that is comparable to hepatic resection (Shiina et al., 2005), however, its therapeutic effect in larger tumors is unsatisfactory owing to the limited coagulative necrotic effect (N’Kontchou et al., 2009; Lencioni, 2010). To overcome this problem, TACE have been introduced especially for the intermediate-stage HCC (Livraghi et al., 2000; McGhana and Dodd, 2001), but complete necrosis is rarely induced by TACE alone due to incomplete embolization or tumor new angiogenesis. So, the combined use of TACE and RFA was tried and showed a synergistic effect that reduces the local progression rate, reduces the recurrence-free survival rate and improves the overall survival in patients with HCC, without significant difference in major complications (Takaki et al, 2009; Min et al., 2013; Wang et al., 2016; Yang et al., 2017). TACE prior to RFA is beneficial for several reasons: a. TACE can improve the effect of RFA thermal coagulation through reducing the cooling effect of hepatic blood flow, b. TACE can lead to ischemic edema, which may enlarge the area of tumor necrosis induced by RFA, c. TACE can reduce the portal venous flow by filling the peripheral portal vein around the tumor, and prevent HCC patients from the portal vein invasion (Seki et al., 2000). Most of cancer therapy researches focus primarily on survival time without significant stress on quality of life (QoL) while nearly 95% of patients with advanced cancer consider a good QoL as important as a long life (Meropol et al., 2003). QoL has been proven to be a valuable parameter and is considered to be as important as overall survival and tumor-free survival for cancer patients (Han et al., 2014) and may be more relevant than length of life, as patients are often more concerned about life-quality than longevity (Qiao et al., 2012). There are multiple questionnaires used to assess the impact of cancer therapy on QoL as a primary or secondary endpoint. Among the most used cancer-specific questionnaires are the European Organization for Research and Treatment of Cancer Quality of Life Questionnaire-Core 30 (EORTC QLQ-C30) (Aaronson et al., 1993) and the EORTC QLQ-HCC18 which is a specific QoL questionnaire for patients with primary liver cancer (Blazeby et al., 2004). According to Barcelona Clinic Liver Cancer (BCLC) HCC staging system, TACE should be considered for patients with intermediate stage of HCC (BCLC-B) (Marrero et al., 2005). The aim of this study was to assess QoL after TACE compared to TACE followed immediately by RFA in patients with unresectable HCC, and to assess tumor response to therapy after these 2 palliative interventions. 

## Materials and Methods

*Patients and Methods*

This is a prospective nonrandomized single center study in consecutive HCC patients presented at Minia University Hospital, Endemic Medicine Department in collaboration with Interventional Radiology Department. Diagnosis of HCC was confirmed by combination of raised α-fetoprotein (AFP) with typical radiological findings with ultrasonography plus triphasic computed tomography or magnetic resonant imaging. Inclusion criteria included: HCC patient with no more than 3 hepatic focal lesions and the patient can complete the questionnaires. Patients were excluded if they had history of other malignancy, encephalopathy, portal vein thrombosis, distant metastasis or cognitive impairment. The study was approved by the institutional ethics committee. Informed consent was obtained in every case.

*Study design*

Every participant filled out the EORTC QLQ-C30 as core questionnaire for cancer disease, and the QLQ-HCC18 questionnaire specially developed for HCC patients one to 3 days before intervention then 2 weeks and 2 months after the intervention. These 2 questionnaires were refilled out after 2 weeks to avoid the bias of post-embolization syndrome occurring within 1 week after intervention. Smaller tumors (≤ 5 cm) were treated by TACE (TACE group, 45 patients) and larger ones (> 5 cm) were treated by TACE followed immediately by RFA at the same sitting (TACE-RFA group, 28 patients). Two hepatology consultants made the therapy decision based on clinical evaluation, imaging studies, comorbidities and BCLC scheme. Lab investigations including liver enzymes, coagulation profile and blood cell count as well as imaging studies were performed before and after interventional therapy. Imaging: abdominal ultrasound was done as a routine followed by contrast enhanced computer tomography (CT) or magnetic resonant imaging (MRI) were obtained before intervention (within two weeks) and follow-up imaging was performed 2 months after intervention.

*TACE Technique*

Percutaneous transarterial access was established through a common femoral artery. Afterwards, celiac and superior mesenteric arteriographs were obtained to confirm portal vein patency and identify accessory arteries. Then, TACE was performed by selectively introducing a catheter into the right or left hepatic artery or a segmental branch of the hepatic artery and injecting a mixture of an iodized oil (4–6 mL, Lipiodol) and chemotherapeutic drugs (mitomycin and doxorubicin) through the main feeding artery under fluoroscopic guidance until the entire tumor became uniformly opacified followed by gelatin sponge particles (Gelfoam). For TACE-RFA group: TACE was performed first, then plain CT was performed immediately and then precise RFA targeted the lesions with poor lipiodol deposition.

*RFA Technique*

A radiofrequency generator working at frequency of 400 KHz, and a RFA electrode needle were used. Under ultrasound guidance, the most optimal puncture site was selected and marked. After local anesthesia infiltration, the electrode needle puncture into the tumor was performed and radio frequency treatment was deployed for 5-10 min with the electrode penetrating the center of tumor, with 1 cm safety margin, caution was considered to avoid large blood vessels or the intrahepatic bile duct. During procedure, ultrasound was used to monitor echo changes in the ablation area, and we adjusted the electrode position until the entire tumor echo was enhanced. Finally, we performed needle tract cauterization to stop bleeding and prevent tract seeding.

*QoL assessment*

EORTC QLQ-C30 is a cancer-specific 30-item questionnaire composed of multiple items and these items are grouped into 9 domains and 6 single items. It incorporates 5 functional domains (physical, role, cognitive, emotional and social), 3 symptom domains (fatigue, pain, and nausea/vomiting) and a global health and QoL domain. The remaining 6 single items assess additional 5 symptoms commonly reported by cancer patients (dyspnea, appetite loss, sleep disturbance, constipation and diarrhea) as well as perceived financial problem (Aaronson et al., 1993). All domains and scales were converted to scores ranging from 0 to 100 according to the scoring manual. A higher score for a functional or global health QoL scale represents a relatively higher/healthier level of functioning or global QoL, while, high scores on the symptom scales/single items indicate worse levels of health state (Fayers, 1999). EORTC QLQ-HCC18 includes 18 multi-item scales. These items are grouped into 6 domains namely fatigue, body image, jaundice, nutrition, pain and fever. The two remaining single items are abdominal swelling and sex life. All scales were grouped and converted to score from 0 to 100 according to the scoring manual; a higher score represents a more severe symptom or problem (Blazeby et al., 2004). QoL was compared using these score changes before and 2 weeks then 2 months after intervention.

*Evaluation of therapeutic efficacy*

The modified response evaluation criteria in solid tumor (mRECIST) were employed for the evaluation of therapeutic efficacy. Based on findings from enhanced MRI or CT before and 2 months after intervention, the therapeutic efficacy was evaluated. Evaluation was performed as follows: a. complete remission (CR): all the target lesions disappeared; b. partial remission (PR): the maximal diameter was reduced by at least 30% as compared to that at baseline; c. disease progression (PD): the maximal diameter increased by 20% as compared to that at baseline, or one or more lesions were observed; d. stable disease (SD): the condition between PR and PD (Lencioni and Llovet, 2010). The efficacy (response rate) was calculated as CR + PR. 

*Statistics*

Statistical analysis was performed using IBM SPSS Statistics (version 20, USA). Statistical significance was compared by Mann–Whitney U test for characteristics on ordinal scale and by X2 for those on interval scale. P value ≤ 0.05 was considered significant

## Results

A total of 110 patients who met the inclusion criteria (BCLC-B) were included in this study. Thirty-seven patients were excluded as they refused/unable to complete the questionnaires or lost during the follow up period. Only 73 patients had completed the QoL questionnaire data and were included for analysis. The median age at diagnosis was 60, the majority were males (80.8%). Almost all patients were post hepatitis C virus (HCV) infection (70 cases), only 3 cases had dual HCV and HBV infection. More than 61% of patients was of Child-Pugh class B. Basic Characteristics of all patients are shown in Table 1. TACE or TACE-RFA were performed smoothly in all the patients with a success rate of 100%. Intra and post-operative severe complications (like severe bleeding or perforation) were not observed. Minor complications like mild to moderate pain, fever, nausea or vomiting had occurred and were insignificantly higher in TACE-RFA group than in TACE group and all disappeared within 2-4 days on symptomatic treatment. Table 2 showed the Mean scores of QLQ-C30 and QLQ-HCC18 scales before intervention. The mean percent of pre-interventional global health status/QoL in TACE-RFA group was significantly lower compared to TACE group (51.2% Vs 64.1%, p = 0.027). Apart from cognitive functioning and social functioning, TACE-RFA group showed significant lower scores in the other functional scales. TACE-RFA group showed significant higher scores for fatigue, nausea/vomiting, pain and appetite loss in the QLQ-C30 symptom scales. Other items of symptom scales of QLQ-C30 scales were higher in TACE-RFA group compared to TACE group but statistically insignificant. Regarding QLQ-HCC18 symptom scales, TACE-RFA group showed significantly higher scores for pain, fatigue, and nutrition problems. In general, TACE-RFA group showed higher scores for symptom scales in both questionnaires. 

Table 3 showed the changes in QoL 2 weeks after intervention: Regarding to pre-/post-changes in EORTC QlQ-C30, TACE-RFA group showed a higher but statistically insignificant decrease in all functional scales compared to TACE group. The mean decrease in global health status/QoL was higher in TACE-RFA group (- 12.1%) compared to TACE group (- 6.3%), which was, however, statistically insignificant (p = 0.411). Also, all symptoms scales showed higher but statistically insignificant increase in TACE-RFA group compared to TACE group except the absolute increase in fatigue which was significantly higher in TACE-RFA group compared to TACE group (+ 23.8% Vs + 13.1%, p = 0.041). For EORTC-HCC18, all symptoms and single item scales showed higher but statistically insignificant increase in TACE-RFA group compared to TACE group. The highest pre-/post changes for TACE group were observed in dyspnea, fatigue, role functioning and social functioning, while the highest pre-/post changes for TACE-RFA group were observed in fatigue, appetite loss, dyspnea, and physical functioning. 

Table 4 showed the changes in QoL 2 months after intervention: generally, there was improvement of all functional scales and symptom scales in both questionnaires but the TACE-RFA group showed significant increase in global health state, physical and social functioning scales and a significant decrease in pain and fatigue regarding the symptom scales. 

**Figure 1:**
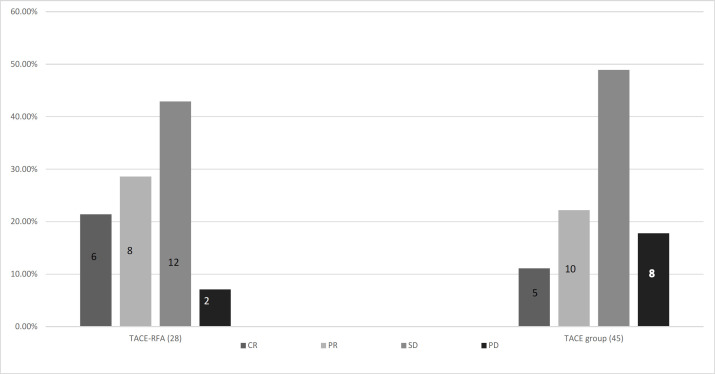
Tumor Response 2 Months after Intervention According to the mRECIST Criteria in Both Groups. TACE-RFA group (28 cases): CR, (6 cases); PR, (8 cases); SD, (12 cases); PD, (2 cases) & TACE group (45 cases); CR, (5 cases); PR, (10 cases); SD, (22 cases); PD, (8 cases)

Tumor response: 2 months after intervention and according to the mRECIST criteria, TACE-RFA group showed better tumor response than TACE group, (CR 21.4, PR 28.6%, SD 42.9%, PD 7.1%), Versus CR11.1%, PR 22.2%, SD 48.9%, PD 17.8% as shown in Figure 1.

**Table 1:** Baseline Characteristics of All Patients in the TACE Group and TACE-RFA Group

	TACE (45 cases)		TACE-RFA (28 cases)		P value
Male/female (No and %)	37/8	82.2% / 17.3%	22/6	78.6% / 21.4 %	0.166
Age in years: mean (Min-Max))	45	58 (49 - 63.6)	28	61.6 (53.6-68.6)	0.617
BMI (kg/m2): mean (Min-Max)	45	23.1 (20.8 - 27.2)	28	22.9 (19.8-26.1)	0.541
Performance state (≤1/ ≥ 2): (No and %)	16/29	35.6%/ 64.4%	7/21	25%/ 75%	0.063
Income (Low/median/High): (No and %)	15/18/12	33.3%/40%/26.7%	12/10/06	42.9%/35.7%/21.4%	0.218
Education (low/middle/high): (No and %)	12/21/12	26.6%/46.7%/26.7	6/12/10	21.4%/42.9%/35.7%	0.492
Child-Pugh class (A/B/C): (No and %)	12/27/6	26.6%/60%/13.4%	6/18/14	21.4%/64.3%/14.3%	0.616
Tumor diameter (cm): mean (Min-Max)	45	3.8 (3.2-5)	28	8.7 (5.2-12.5)	0.571
a -fetoprotein (ng/mL): mean (Min-Max)	45	168 (86-398)	28	231 (115-482)	0.321
mRECIST (mm): mean (Min-Max)	45	40 (32-50)	28	65 (55-148)	0.062

Variables on an ordinal scale were tested by Mann–Whitney U test and those on an interval scale by X^2^. Significance when p ≤ 0.05.

**Table 2:** Mean Pre-Interventional Score Percent for Each QoL Domain of the QlQ-C30 and the HCC18 Questionnaires in the TACE Group and TACE-RFA Group

Variable	TACE (45 cases)	TACE-RFA (28 cases)	P value
QoL-C30: Mean pre-interventional score:			
1. Global health status/QoL:	64.1	51.2	0.027
2. Functional scales:			
Physical functioning	86.9	63.1	0.003
Role functioning	85.2	62.9	0.002
Emotional functioning	72.5	59.5	0.019
Cognitive functioning	81.8	78.6	0.064
Social functioning	84.1	77.3	0.091
3. Symptom scales/items			
Fatigue	25.6	55.9	0.001
Nausea/vomiting	6.3	17.1	0.003
Pain	27.3	48.9	0.028
Dyspnea	31.5	39.5	0.061
Insomnia	52.6	60.3	0.411
Appetite loss	23.1	38.7	0.041
Constipation	15.7	18.5	0.812
Diarrhea	9.8	9.3	0.991
Financial difficulties	15.1	21.5	0.391
QLQ-HCC 18			
4. Symptom scales/items			
Fatigue	27.1	51.4	0.011
Body image	19.3	26.9	0.512
Jaundice	9.3	15.3	0.438
Nutrition problems	13.9	22.3	0.025
Pain	12.3	34.8	0.042
Fever	9.2	12.5	0.563
5. Single items			
Abdominal swelling	11.9	21.9	0.072
Sexual life	16.5	21.4	0.345

Mann-Whitney U test was used, and P value was considered significant when ≤ 0.05

**Table 3:** Two Weeks after Intervention; the Mean change in the score percent of each QoL domain of the QlQ-C30 and the HCC 18 questionnaires in the TACE group and TACE-RFA group compared to baseline

Variable	TACE (45)	TACE-RFA (28)	P value
QoL-C30			
1. Global health status/QoL:	-6.3	-12.1	0.411
2. Functional scales:			
Physical functioning	-10.3	-17.9	0.328
Role functioning	-13.3	-15.6	0.214
Emotional functioning	-7.1	-13.5	0.661
Cognitive functioning	-6.9	-8.4	0.761
Social functioning	-13.9	-15.1	0.591
3. Symptom scales/items			
Fatigue	13.1	23.8	0.041
Nausea/vomiting	8.6	11.1	0.411
Pain	11.5	13.1	0.419
Dyspnea	14.6	16.7	0.328
Insomnia	11.1	12.9	0.213
Appetite loss	11.6	16.9	0.612
Constipation	9.8	10.2	0.619
Diarrhea	3.2	4.3	0.491
Financial difficulties	8.3	9.9	0.415
QLQ-HCC 18			
4. Symptom scales/items			
Fatigue	11.2	16.9	0.681
Body image	15.3	12.3	0.405
Jaundice	1.1	3.55	0.415
Nutrition problems	12.5	14.1	0.319
Pain	12.5	9.7	0.218
Fever	10.1	7.6	0.551
5. Single items			
Abdominal swelling	12.7	14.2	0.617
Sexual life	13.4	11.6	0.662

**Table 4:** Two Months after Intervention; the Mean change in the score percent of each QoL domain of the QlQ-C30 and the HCC18 questionnaires in the TACE group and TACE-RFA group compared to baseline

Variable	TACE (45)	TACE-RFA (28)	P value
QoL-C30			
1. Global health status/QoL:	+11.2	+19.1	0.023
2. Functional scales:			
Physical functioning	+13.6	+22.2	0.031
Role functioning	+18.9	+21.5	0.077
Emotional functioning	+9.1	+12.8	0.059
Cognitive functioning	+12.5	+13.9	0.832
Social functioning	+11.6	+23.5	0.011
3. Symptom scales/items			
Fatigue	-10.9	-18.7	0.021
Nausea/vomiting	-16.4	-19.3	0.134
Pain	-12.6	-22.1	0.044
Dyspnea	-15.5	-18.9	0.254
Insomnia	-15.8	-18.5	0.347
Anorexia	-12.4	-10.4	0.148
Constipation	-14.7	-12.3	0.527
Diarrhea	-12.5	-16.2	0.173
Financial difficulties	-18.8	-19.6	0.356
QLQ-HCC 18			
4- Symptom scales/items			
Fatigue	-11.5	-18.9	0.033
Body image	-19.1	-18.2	0.841
Jaundice	-3.3	-9.5	0.125
Nutrition problems	-14.6	-15.5	0.272
Pain	-15.2	-23.3	0.032
Fever	-15.1	-25.2	0.152
5. Single items			
Abdominal swelling	-12.7	-18.6	0.145
Sexual life	-18.1	-19.7	0.771

## Discussion

QoL is defined as people’s perceptions of their position in life in the context of the culture and value systems in which they live, and in relation to their goals, expectations, standards, and concerns (Fayers, 1999). With the aim of improving or at least maintaining the QoL of HCC patients, QoL has been increasingly used as an outcome measure of different treatment modalities. This study was designed to compare the changes in QoL in patients with unresectable HCC subjected to TACE or TACE plus RFA before and after the intervention. TACE-RFA group of patients had a significant lower score of initial global health status, role functioning, physical functioning, emotional functioning and a higher score of pain, fatigue, nausea/vomiting and appetite loss. Two weeks after intervention, TACE-RFA leads to higher impairment of QoL including global health status, functional scales and symptoms scales compared to TACE alone however it was statistically insignificant. Considering that fatigue, abdominal pain, fever, ascites, and/or jaundice are well-known manifestations of advanced HCC, TACE-RFA resulted in a higher increase in fatigue, fever, pain and abdominal swelling, and more deterioration of jaundice when compared to TACE alone, nevertheless, statistical significance for all of these changes was not reached except for fatigue (P= 0.041), This might suggest that both modalities have the same tolerability without significant effect on the changes in QoL and that TACE-RFA does not significantly impair QoL even in HCC patients with higher tumor burden. Two months after intervention, our data of QoL showed that there was improvement in all functional scores as well as symptoms scores in both groups, however, the TACE–RFA group showed significant increase in global health state, physical and social functioning scales (P= 0,023, 0.031, and 0.011 respectively) and a significant decrease in fatigue and pain regarding the symptom scales (P= 0.033 and 0.032 respectively) and this is consistent with previous studies done by Bloomston et al, 2002, and Wang et al., (2007). 

TACE or TACE-RFA as a palliative treatment for unresectable HCC induces necrosis in the tumor largely due to the embolization and/or the radiofrequency effects without a volume reduction. mRECIST criteria are therefore more appropriate to measure therapeutic effect in these tumors (Shim et al., 2012). In our study, TACE-RFA showed a better mRECIST response rates (CR 21.4%, PR 28.6%, SD 42.9%, PD 7.1%), compared to TACE alone (CR 11.1%, PR 22.2%, SD 48.9%, PD 17.8%), and this is in agreement with Yang et al., (2017), who concluded that TACE+RFA simultaneously is an effective, safe, and precise technique for the treatment of large HCC and could improve the focal control rate and survival rate. Wang et al., (2013), retrospectively studied 18 patients with large solitary HCC > 5 cm who were subjected to immediate combination therapy of TACE+RFA with the clinical efficiency was 100% in all patients, (17 cases of CR and 1 case of PR), and the estimated overall survival rate at 6, 12, and 18 months was 100%, with no major complications observed. Bhangoo et al, 2015, summarized mRECIST CR, PR and SD rate as a ‘‘clinical benefit’’ in a study analyzing transarterial radio embolization (TARE) for patients with unresectable HCC and this study showed that this ‘‘clinical benefit’’ was obtained in 74% of the patients undergoing TARE. This ‘clinical benefit’ (mRECIST CR, PR and SD rate) was reported to be 100%, 97%, 95.5%, and 90.1% after 1, 3, 6, and 12 months respectively in a study evaluating TACE+RFA in the same sitting for treatment of large unresectable HCC (Yang et al, 2017). In our study, this ‘clinical benefit’ was 26/28(92.9%) for TACE-RFA group compared to 37/45 (82.2%) for TACE group after 2 months follow up, and this suggest that immediate TACE-RFA induces better tumor response (better clinical benefit) when compared to TACE alone even with larger tumor size. In other hand, Hyukjoon et al, 2018, reported that combined therapy using TACE immediately followed by RFA is a safe and effective treatment in early stage HCCs (BCLC stage 0 or A) as it provides less local tumor progression and longer time to progression than TACE alone (P=0.013 and 0.037 respectively) with no major complication in either group.

*Study limitations*

One of the limitations is the short follow-up period and the small number of participants treated in only one center. Also, the study is not randomized, and lastly, this study did not assess QoL in correlation with the therapeutic response. Future larger scale studies using same QoL questionnaires evaluated at multiple time points during a longer duration of follow-up is warranted.

I conclusion, neither TACE nor TACE-RFA showed a significant decrease in QoL in patients with unresectable HCC two weeks after intervention. However, two months after intervention; TACE-RFA showed significant improvement in global health score, social and physical functioning scores, as well as significant improvement in pain and fatigue compared to TACE monotherapy. This might suggest that simultaneous TACE-RFA is safe and effective palliative option for unresectable HCC and more favorable than TACE monotherapy regarding QoL or tumor response to therapy. Simultaneous TACE-RFA should be encouraged to be used by physicians caring for HCC patients.

## Author Contribution Statement

Yasser Fouad and Taha M Hassanin: the concept and design of the study. Wael Abdelghany and Alshymaa Hassanin were responsible for data acquisition. Naglaa Farag: performed the laboratory part of the study. Mohamad Eisawy performed TACE or TACE-RFA technique. Taha M Hassanin, Yasser Fouad were responsible for statistical analysis, interpreted the results and drafted the manuscript. All authors critically revised the manuscript, approved the final version to be published, and agreed to be accountable for all aspects of the work.

## References

[B1] Aaronson NK, Ahmedzai S, Bergman B (1993). The European Organization for Research and Treatment of Cancer QLQ-C30: a quality-of-life instrument for use in international clinical trials in oncology. J Natl Cancer Inst.

[B2] Bhangoo MS, Karnani DR, Hein PN (2015). Radioembolization with yttrium-90 microspheres for patients with unresectable hepatocellular carcinoma. J Gastrointest Oncol.

[B3] Blazeby J, Currie E, Zee BCY (2004). Development of a questionnaire module to supplement the EORTC QLQ-C30 to assess quality of life in patients with hepatocellular carcinoma, the EORTC QLQ-HCC18. Eur J Cancer.

[B4] Bloomston M, Binitie O, Fraiji E (2002). Transcatheter arterial chemoembolization with or without radiofrequency ablation in the management of patients with advanced hepatic malignancy. Am Surg.

[B6] Han KT, Park EC, Kim SJ (2014). Factors affecting the quality of life of Korean cancer survivors who return to the workplace. Asian Pac J Cancer Prev.

[B7] Hyukjoon Lee, Chang JY, Nak JS, Jeong SH, Kim JW (2018). Comparison of combined therapy using conventional chemoembolization and radiofrequency ablation versus conventional chemoembolization for ultrasound-invisible early-Stage Hepatocellular Carcinoma (Barcelona Clinic Liver cancer stage 0 or A). Korean J Radiol.

[B8] Lencioni R (2010). Loco-regional treatment of hepatocellular carcinoma. Hepatology.

[B9] Lencioni R, Llovet JM (2010). Modified RECIST (mRECIST) assessment for hepatocellular carcinoma. Semin Liver Dis.

[B10] Livraghi T, Goldberg SN, Lazzaroni S (2000). Hepatocellular carcinoma: radio-frequency ablation of medium and large lesions. Radiology.

[B11] Marrero JA, Fontana RJ, Barrat A (2005). Prognosis of hepatocellular carcinoma: comparison of 7 staging systems in an American cohort. Hepatol.

[B12] McGhana JP, Dodd GD (2001). Radiofrequency ablation of the liver: current status. AJR Am J Roentgenol.

[B13] Meropol NJ, Weinfurt KP, Burnett CB (2003). Perceptions of patients and physicians regarding phase I cancer clinical trials: implications for physician-patient communication. J Clin Oncol.

[B14] Min JH, Lee MW, Cha DI (2013). Radiofrequency ablation combined with chemoembolization for intermediate-sized (3-5 cm) hepatocellular carcinomas under dual guidance of biplane fluoroscopy and ultrasonography. Korean J Radiol.

[B15] N’Kontchou G, Mahamoudi A, Aout M (2009). Radiofrequency ablation of hepatocellular carcinoma: long-term results and prognostic factors in 235 Western patients with cirrhosis. Hepatology.

[B16] Qiao CX, Zhai XF, Ling CQ (2012). Health-related quality of life evaluated by tumor node metastasis staging system in patients with hepatocellular carcinoma. World J Gastroenterol.

[B17] Raoul JL, Forner A, Bolondi L (2019). Updated use of TACE for hepatocellular carcinoma treatment: How and when to use it based on clinical evidence. Cancer Treat Rev.

[B18] Sangiovanni A, Del Ninno E, Fasani P (2004). Increased survival of cirrhotic patients with a hepatocellular carcinoma detected during surveillance. Gastroenterology.

[B19] Seki T, Tamai T, Nakagawa T (2000). Combination therapy with transcatheter arterial chemoembolization and percutaneous microwave coagulation therapy for hepatocellular carcinoma. Cancer.

[B20] Shiina S, Teratani T, Obi S, Sato S (2005). A randomized controlled trial of radiofrequency ablation with ethanol injection for small hepatocellular carcinoma. Gastroenterology.

[B21] Shim J, Lee H, Kim S (2012). Which response criteria best help predict survival of patients with hepatocellular carcinoma following chemoembolization? A validation study of old and new models. Radiology.

[B22] Shipra G, Sapna K, Renuka I (2014). Quality of life and hepatocellular carcinoma. J Gastrointest Oncol.

[B23] Takaki H, Yamakado K, Uraki J (2009). Radiofrequency ablation combined with chemoembolization for the treatment of hepatocellular carcinomas larger than 5 cm. J Vasc Interv Radiol.

[B24] Torre LA, Bray F, Siegel RL (2015). Global cancer statistics. CA Cancer J Clin.

[B25] Wang Xin, Yanan Hu, Mudan Ren (2016). Efficacy and safety of radiofrequency ablation combined with transcatheter arterial chemoembolization for hepatocellular carcinomas compared with radiofrequency ablation alone: A Time-to-Event Meta-Analysis. Korean J Radiol.

[B26] Wang YB, Chen MH, Kun Y (2007). Quality of life after radiofrequency ablation combined with transcatheter arterial chemoembolization for hepatocellular carcinoma: comparison with transcatheter arterial chemoembolization alone. Qual Life Res.

[B27] Wang ZJ, Wang MQ, Feng D (2013). Transcatheter arterial chemoembolization followed by immediate radiofrequency ablation for large solitary hepatocellular carcinomas. World J Gastroenterol.

[B28] Yang TZ, Yong FL, Wang MQ, Xian XC (2017). Transcatheter arterial chemoembolization combined with simultaneous computed tomographyguided radiofrequency ablation for large hepatocellular carcinomas. Chin Med J.

